
JOURNAL INFORMATION
==============================
NLM Title Abbreviation: Wirtschaftsdienst
Journal ID: Wirtschaftsdienst
NLM Journal ID: 101086612

Wirtschaftsdienst (Hamburg, Germany : 1949)

ISSN: 0043-6275

ARTICLE INFORMATION
==============================
PMCID: PMC7765693
PMID: 33390622
DOI: 10.1007/s10273-020-2798-9
Article ID: 2798
Article version: 1
Subjects: Zeitgespräch

Der amerikanische gordische Knoten

Komlos John John.Komlos@gmx.de
1
Schubert Hermann hermann.schubert@ism.de
2
1 grid.5252.0 0000 0004 1936 973X Wirtschaftsgeschichte- und theorie, LMU München, Ludwigstr. 33, 80539 München, Deutschland
2 grid.466363.2 0000 0004 0418 4417 Economics & Quantitative Methods Depart., International School of Management, Olgastraße 86, 70180 Stuttgart, Deutschland
Electronic publication date: 2020 Dec 26
Print publication date: 2020
Volume: 100
Issue: 12
First page: 923
Last page: 927
Copyright: © Der/die Autor(en) 2020
License: Dieser Artikel wird unter der Creative Commons Namensnennung 4.0 International Lizenz (https://creativecommons.org/licenses/by/4.0/deed.de) veröffentlicht.
Open Access wird durch die ZBW — Leibniz-Informationszentrum Wirtschaft gefördert.
License URL: https://creativecommons.org/licenses/by/4.0/

issue-copyright-statement: © The Author(s) 2020

==============================

Weite Teile der Welt sind erleichtert nach der Wahl von Joe Biden, jedoch sind die Herausforderungen für die kommende Regierung gewaltig und erscheinen unüberwindbar (Komlos und Schubert, 2020). Betrachten wir die Lage zunächst aus der Vogelperspektive. Die westliche Zivilisation befindet sich inmitten einer Informationsrevolution an einem Scheideweg und entwickelt sich zu einer postindustriellen wissensbasierten Ökonomie. Wie sämtliche historische Erfahrungen belegen, gestalten sich solche umfassenden wirtschaftlichen Transformationen immer kompliziert, niemals reibungslos und selten konfliktfrei. Der Übergang vom Feudalismus zum Kapitalismus war kein Kinderspiel. Immerhin hatte Frankreich während des Übergangs vier Revolutionen, während England im 17. Jahrhundert zwei große Umwälzungen erlebte, bei denen ein König enthauptet und ein anderer durch Machenschaften abgesetzt wurde, die einem modernen Staatsstreich ähnelten. Insofern solche Transformationen die politische, soziale und wirtschaftliche Ordnung neu definieren und herkömmliche Hierarchien infrage stellen, sollte es uns wenig überraschen, wenn daraus Konflikte entstehen. Es ist ein dorniger Pfad, Macht oder sonstige Privilegien aufzugeben (Sandel, 2018). Dies erkannten bereits die alten Griechen (Thukydides, um 411 v. Chr).

Während das Gesamtbild uns hilft, den Kontext der aktuellen Situation zu verstehen, sollte man sich vor Augen halten, dass die USA in politischen, kulturellen, sozialen und wirtschaftlichen Bereichen mit zahlreichen, sich gegenseitig beeinflussenden Problemen konfrontiert sind. Die wichtigsten 20 Herausforderungen, vor denen die neu gewählte Regierung von Joe Biden gestellt wird, sollen im Folgenden dargestellt werden.

## Politische Dysfunktion als fester Bestandteil des US-amerikanischen Systems

1. Das in der US-amerikanischen *Verfassung* verankerte Wahlsystem ist anachronistisch. Eine Errungenschaft der Aufklärung, dass der Gemeinwille durch die Abstimmung freier Bürger*innen ermittelt, zu Gesetzen wird und für alle bindend ist, gilt in den USA nicht (Rousseau, 1762). Obwohl Al Gore in der Präsidentschaftswahl von 2000 im ganzen Land mehr Stimmen erhielt als George W. Bush, ging dieser als Sieger der Wahl hervor, da Letzterer mehr Wahlleute gewinnen konnte. Ebenso verhielt es sich zwischen Hillary Clinton und Donald Trump bei der Präsidentschaftswahl 2016. Trotz eines Vorsprungs von 3 Mio. Stimmen verlor sie die Wahl. Demzufolge sind die USA trotz Wahlen keine funktionierende Demokratie, da einige Stimmen mehr als andere zählen und die gesamte Wahl auch gegen die Mehrheit des wählenden Volks entschieden werden kann. In den Präsidentschaftswahlen 2020 entscheiden 300.000 kalifornische Wähler*innen für eine Wahlperson, die im Dezember im Electoral College offiziell den Präsidenten wählt, während in den Bundesstaaten Montana, Wyoming, South und North Dakota rund 140.000 Wähler*innen eine Wahlperson wählen. Die Stimmen der Bürger*innen dieser vier Staaten sind also doppelt so wichtig wie jene der Bürger*innen Kaliforniens. Solche Gewichtungsschemata erklären ebenfalls den nicht repräsentativen Charakter des Senats. Dort hat Kalifornien zwei Vertretungen, während die weniger bevölkerten 22 Staaten mit einer Gesamtbevölkerung von Kalifornien 44 Senator*innen entsenden. Eine funktionierende Demokratie sollte sicherstellen, dass der allgemeine Wille der Bevölkerung die Herrschaft eines Landes bestimmt, was so aber in den USA nicht gegeben ist (Sandel, 2018).

2. Mit der großen Rolle des Geldes in der Politik und dem durchschnittlichen Vermögen der obersten 1 % der Haushalte von 25 Mio. US-$ haben sich die USA in eine *Plutokratie* verwandelt (Page und Gilens, 2017). Mit der Entscheidung des Obersten Gerichtshofs, politische Aktionskomitees, die sogenannten Super-PACs, für die Wahlkampffinanzierung zuzulassen, gewann die Plutokratie entscheidend an politischem Einfluss. Denn anders als bei der direkten Finanzierung von Präsidentschaftskandidat*innen unterliegen die Super-PACs, die den Wahlkampf einer/s Kandidat*in unterstützen und aus eigenen Mitteln finanzieren, keinerlei Beschränkungen, wie viel Geld sie von einzelnen Spender*innen erhalten können. Der politische Einfluss der Plutokratie, eine Person zu unterstützen oder zu verhindern, wächst dadurch ungemein. Trump hätte sich in den Vorwahlen der Republikaner 2016 mit hoher Wahrscheinlichkeit nicht durchsetzen können, wenn er nicht auf eigene finanzielle Mittel hätte zurückgreifen können. Nach Schätzungen des unabhängigen Center for Responsive Politics (CRP) haben beide Parteien im Wahlkampf um die Präsidentschaft zwischen Biden und Trump 11 Mrd. US-$ ausgegeben.

3. Der *militärisch-industrielle Komplex* hat einen starken Einfluss auf den politischen Prozess. Die jährlichen Militärausgaben in Höhe von 720 Mrd. US-$ belasten den Bundeshaushalt enorm und entziehen der Zivilbevölkerung einen beträchtlichen Teil des Steueraufkommens. Die militärischen Abenteuer im Irak und in Afghanistan haben Billionen US-Dollar gekostet.

## Kaum zu überwindende kulturelle Herausforderungen

4. Seit Reagan ist der Neoliberalismus die dominante *Ideologie* der USA. Unzählige, von der Plutokratie finanzierte Thinktanks verbreiten seit Jahrzehnten erfolgreich die Überlegenheit von Märkten gegenüber dem Staatshandeln und verleugnen gezielt seine Schwächen und sind so tief in die Populärkultur eingedrungen, dass es schwierig wird, die Bevölkerung davon zu überzeugen, dass nicht der Staat, der am wenigsten regiert, auch der beste sei (Komlos, 2015). Für die Apologet*innen des Marktfundamentalismus ist es einfach, jedes sozialpolitische Programm fortschrittlicher Politik, das den Zustand armer Bevölkerungsschichten verbessern möchte, als Sozialismus zu diffamieren.

## Tief verwurzelte soziale Herausforderungen

5. Struktureller *Rassismus* ist ein nicht zu übersehendes Phänomen in der US-amerikanischen Gesellschaft. Der Mangel an Arbeitsplätzen, das unterfinanzierte öffentliche Bildungssystem und das begrenzte Sicherheitsnetz belasten die Lebensumstände der Minderheiten in den USA. Die Arbeitslosenquote unter Minderheiten ist etwa doppelt so hoch wie in der weißen Bevölkerung. Afroamerikaner*innen sind 1,8-mal häufiger arm als es ihrem Anteil an der Bevölkerung entspricht, und ihr jährliches durchschnittliches Haushaltseinkommen lag 2016 um 24.600 US-$ unter dem der Weißen. Sie werden wegen ihrer Armut diskriminiert und haben häufig keinen Zugang zum Gesundheitssystem. In den USA herrscht eine tiefe Kluft zwischen der Qualität von Schulen in benachteiligten Gegenden, in denen häufig Minderheiten wohnen, und den Schulen der reichen weißen Nachbarn. Die Ursache dafür ist, dass sich die Schulen in den USA aus den lokalen Grundsteuern finanzieren und die Schulen in armen Gegenden chronisch unterfinanziert sind. Wer jedoch keine gute Ausbildung genießt, bekommt keinen guten Job, keine Krankenversicherung und bleibt arm. Die Sterblichkeitsrate unter Afroamerikaner*innen von 114 pro 100.000 ist praktisch doppelt so hoch wie in der weißen Bevölkerung (62 pro 100.000) (APM Research Lab Staff, 2020).

6. Es ist allgemein anerkannt, dass die Verteilung von Einkommen und Vermögen in den USA sehr *ungleich* ist und die Hälfte der Bevölkerung daran leidet. Das obere eine Prozent der Gesellschaft verdient 20 % des Nationaleinkommens. Durch die Rettungsaktionen des Finanzsystems 2008 wurden nahezu alle hochdotierten Bankmanager*innen vom Abstieg in die Mittelschicht verschont. Dick Fuld, der letzte Vorstand und Chief Executive Officer der Investmentbank Lehman, war für die Insolvenz von Lehmans mitverantwortlich. Sein Vermögen wird aktuell auf 250 Mio. US-$ geschätzt. Angelo Mozilo, der Gründer und CEO des größten US-amerikanischen Hypothekenanbieters Countrywide Financial Corporation, verfügt über ein Vermögen von 600 Mio. US-$, während 8 Mio. Familien während der Finanzkrise ihre Häuser verloren. Solche asymmetrischen Rettungsaktionen weisen auf die Macht der Oligarchie hin und haben nicht zuletzt 2016 die Wahl von Trump ermöglicht und den mit ihm aufflammenden Populismus angefeuert (Komlos und Schubert, 2019).

7. Die Qualität des primären und sekundären *Bildungssystems* ist mittelmäßig in den USA und wird den Herausforderungen der IT-Revolution für zukünftige Generationen nicht gerecht. Ein großer Anteil der armen Bevölkerung wird sich weiterhin keine College-Ausbildung leisten können und wird insbesondere in einer Wissensgesellschaft auch weiterhin abgehängt bleiben. So erklärt sich auch die erhebliche Überschussnachfrage nach qualifizierten IT-Fachleuten in den USA. Das US-amerikanische Bildungssystem bringt zu wenige IT-Fachleute hervor. Für den Staat stellt es sich offenbar als kostengünstiger dar, im Ausland ausgebildete Fachleute zu rekrutieren, als das heimische Bildungssystem zu reformieren.

8. Die *Lebenserwartung* der US-amerikanischen Gesellschaft fällt seit 2014, bereits seit sechs Jahren vor der Pandemie. Die 150.000 Menschen, die an „Todesfällen aus Verzweiflung“ sterben, vermitteln einen Hinweis auf die desolate Lage der Menschen in den unteren Einkommensklassen (Case und Deaton, 2020). So hohe Zahlen sind in Gesellschaften mit einem funktionierenden Wirtschafts- und Sozialsystem nirgendwo zu beobachten.

## Eine Volkswirtschaft gerät aus dem Gleichgewicht

9. Das *anämische Wirtschaftswachstum* ist zur Normalität im 21. Jahrhundert geworden und wird sich wahrscheinlich auch nicht ändern. Das Pro-Kopf-Wachstum erreichte zwischen 2000 und 2019 1,1 % und liegt deutlich unter dem Durchschnittswert von 2,1 % während der letzten drei Dekaden im 20. Jahrhundert.[1] Die USA haben nach der Finanzkrise 2008 für rund 1 Billion US-$ weniger Waren produziert als vor der Krise. Aber wie Larry Summers betont, gab es bereits vor der Krise keine Anzeichen für eine überhitzte Wirtschaft, trotz der expansiven Geldpolitik, der Explosion der Schulden und der „fahrlässigen Kreditvergabe“. Verbraucherpreise, Löhne und Produktion wuchsen nicht überdurchschnittlich (Summers, 2015). Stattdessen schieden Millionen von Menschen aufgrund mangelnder Möglichkeiten aus dem Erwerbsleben aus oder fanden nur schlechtbezahlte Teilzeitstellen oder befristete Jobs. Dies veranlasste Summers zu der These der säkularen Stagnation: Aufgrund der unzureichenden Gesamtnachfrage wird die postindustrielle Dienstleistungswirtschaft auf absehbare Zeit unter Wachstumsschwächen leiden. Daher besteht kein Grund zu der Annahme, dass ein beschleunigtes Wirtschaftswachstum die notwendigen Ressourcen freisetzen könnte, um den immer offener ausgetragenen Verteilungskonflikt in der US-amerikanischen Gesellschaft zu entschärfen.

10. Das anämische Wirtschaftswachstum ist teilweise auf die *Verlangsamung des Produktivitätsanstiegs* zurückzuführen, und es besteht kein Grund zur Annahme, dass sich dieser Prozess nicht fortsetzen wird (Gordon, 2016). Die Gesamtfaktorproduktivität stieg zwischen 1988 und 2007 jährlich um 1 % und halbierte sich zwischen 2007 und 2019 auf 0,5 % (Federal Reserve Bank, 2020b).[2] Das bedeutet, dass Innovation und Unternehmertum nicht länger als Garant für Wirtschaftswachstum gelten können.

11. Das *endemische Haushaltsdefizit* impliziert, dass die Regierung nur über wenige Möglichkeiten für die Finanzierung von Großprojekten, die eines der strukturellen Probleme der Wirtschaft mindern könnten,

1 Vgl. 4. Quartal im Verhältnis zum 4. Quartal; vgl. Federal Reserve Bank of St. Louis (Federal Reserve Bank, 2020a).

2 Die Arbeitsproduktivität fiel in der gleichen Periode von 2,4 % auf 1,4 %; vgl. Federal Reserve Bank (2020c).

verfügt, selbst wenn sie mit einer dazu notwendigen politischen Mehrheit ausgestattet wäre (Rogoff, 2019). Dies bedeutet auch, dass die Zinszahlungen für die Staatsverschuldung weiter ansteigen werden. Im Jahr 2018 betrug der durchschnittliche Zinssatz 2,3 %. Sollte es jedoch zu einem Zinsanstieg kommen, auch wenn gegenwärtig wenig dafür spricht, würde die Schuldenlast prekär werden (DeSilver, 2017). Umso mehr, da die Staatsverschuldungsquote infolge der Coronakrise von 103 % auf 135 % angestiegen ist (Federal Reserve Bank, 2020d).

12. Die *Verschuldung des privaten Sektors* erreicht im Jahr 2019 ebenfalls ein gefährliches Niveau. Die Verschuldung von Kreditkartenbesitzer*innen liegt bei 0,9 Billionen US-$ und mehr als 40 Mio. Menschen müssen Studienkredite in Höhe von 1,6 Billionen US-$ tilgen. All diese verschuldeten jungen Menschen werden weniger konsumieren und die Gesamtnachfrage dämpfen.

13. Die *technologische Arbeitslosigkeit* wird eine dauerhafte Bedrohung sein, es sei denn, es werden wie in den 1930er Jahren die geleisteten Arbeitsstunden pro Arbeitnehmer*in reduziert und alle Einkommen und Arbeitsplätze garantiert. Der vermehrte Einsatz von künstlicher Intelligenz bedeutet, dass die Beschäftigung zunehmend vom Wachstum des BIP entkoppelt wird. Minderqualifizierte Menschen werden durch die Automatisierung und endemische Unterbeschäftigung zunehmend überflüssig (Brynjolfsson und McAfee, 2014). Die Diskussion über die Einführung eines Grundeinkommens und arbeitsfördernde Programme wird auch in den USA auf einem der vorderen Plätze der politischen Agenda stehen (Komlos, 2019).

14. Die offiziell veröffentlichte *Arbeitslosenquote* unterschätzt die tatsächliche ökonomische Arbeitslosenquote um ca. 50 %, da ihre Berechnungsgrundlage zahlreiche Arbeitslose nicht berücksichtigt. Noch vor der Pandemie wurden 4,9 Mio. Arbeitslose nicht in den offiziellen Statistiken geführt, weil sie den engen Kriterien von Arbeitslosigkeit nicht entsprachen, und weitere 4,3 Mio. Menschen arbeiteten unfreiwillig in Teilzeit, weil sie keine Vollzeitbeschäftigung finden (Federal Reserve Bank, 2020e, 2020f). Die ökonomische Arbeitslosenquote lag 2019 bei 7 % und nicht bei den vielbeachteten 3,5 %, wie es die offiziellen Zahlen suggerierten (Federal Reserve Bank, 2020g, 2020h). Unter Donald Trump setzte kein Jobwunder ein.

15. Stagnierende oder *sinkende Löhne* sind frustrierend und führen zu Verteilungskonflikten, die wiederum den weißen Nationalismus anfachen. Erstaunlicherweise lag der Realmedianlohn der Männer, die Ende 2019 Vollzeit beschäftigt waren, immer noch auf dem Niveau von 1979 (Federal Reserve Bank, 2020i). Die monatlichen Medianeinkommen der Frauen stiegen zwischen 2004 und 2019 um lediglich 114 US-$ (Federal Reserve Bank, 2020j, 2020k).

16. Die *Pandemie* von 2020 ist ein unwahrscheinliches Ereignis mit großer Auswirkung. Solche Ereignisse werden oft als „schwarzer Schwan“ bezeichnet (Taleb, 2007). Es traf mit voller Wucht auf eine fragile Wirtschaft und offenbarte schonungslos die Notwendigkeit grundlegender Reformen zu einem gerechteren und robusteren sozioökonomischen System. Solche unvorhergesehenen Katastrophen sind im 21. Jahrhundert in immer kürzeren Abständen von fünf Jahren aufgetreten: die Dotcom-Blase von 2000, 9/11, die Finanzkrise von 2008 und aktuell die Pandemie. Flankiert werden diese Ereignisse von den immer deutlicher zum Vorschein kommenden Auswirkungen des Klimawandels, der die Häufigkeit von Waldbränden und Hurrikans in bisher ungekannte Höhen treibt. Solche unvorhergesehenen Ereignisse treten so regelmäßig auf, dass wir ihre Bedrohungen viel ernster nehmen sollten als bisher.

17. Ein *endemisches Handelsdefizit* in Höhe von 578 Mrd. US-$ 2019 bedeutet, dass die USA weiterhin Arbeitsplätze exportieren werden, die einen integrativen Arbeitsmarkt behindern werden (U.S. International Trade in Goods and Services FT 900, 2020).

18. *Globalisierung* bedeutet, dass Arbeitende auf der ganzen Welt miteinander konkurrieren. Die US-amerikanischen Arbeitskräfte befinden sich aufgrund der fehlenden institutionellen Unterstützung durch Gewerkschaften und ihrer begrenzten Ausbildungschancen im Nachteil beim Konkurrieren um Jobs in einer postmodernen Wissensgesellschaft.

19. Die *Infrastruktur*, das Lebenselixier jeder Volkswirtschaft, befindet sich in den USA in einem desolaten Zustand und wird das Wirtschaftswachstum weiterhin belasten. Der kommenden Regierung fehlen nach den Steuererleichterungen die notwendigen Mittel, um Investitionen in die Infrastruktur zu finanzieren, um Schlaglöcher auszubessern, Brücken zu warten oder Gebäude zu sanieren.

20. Die US-Wirtschaft hat sich in einen *Rettungskapitalismus* verwandelt, der durch die Geldschöpfung der US-amerikanischen Zentralbank unterstützt wird. Die Federal Reserve Bank hat in den vergangenen Jahren die Wertpapierkäufe intensiviert. Das hat die Bilanzsumme zwischen 2008 und 2020 von 0,8 auf 7,1 Billionen US-$ anschwellen lassen, was einer Steigerung um den Faktor 9 entspricht. Es stellt sich die Frage, wie lange ein solcher Rettungskapitalismus noch funktionieren kann.

## Fazit

Donald Trump prahlte vor der Rezession mit „einem beispiellosen Wirtschaftsboom“ und behauptete, die US-amerikanische Volkswirtschaft sei „die heißeste Wirtschaft überhaupt“ und würde den „Neid der Welt“ erregen, da „in den Vereinigten Staaten ein Wirtschaftswunder stattfände“ (Trump, 2019/2020). Dabei verwechselte er anscheinend ein potemkinsches Dorf mit der Realität (Sachs, 2019). Nach den oben genannten Erkenntnissen befand sich die Struktur der Wirtschaft im Wesentlichen in einem permanenten Ungleichgewicht. Der drohende politische Stillstand aufgrund der Mehrheit der republikanischen Partei im Senat lässt starke Zweifel aufkommen, dass die dargestellten 20 Herausforderungen gelöst werden könnten. Es spricht deshalb wenig dafür, dass die US-amerikanische Gesellschaft in Zukunft sozial integrativer sein wird als bisher. Aus diesen Gründen ist Skepsis angebracht, dass Biden diesen verschlungenen gordischen Knoten durchschlagen und die Wirtschaft so reformieren wird, dass sie den Bedürfnissen und Bestrebungen der US-amerikanischen Bevölkerung gerecht wird.

Es sollte jedoch angemerkt werden, dass dies nicht Bidens Schuld ist. Der US-amerikanische gordische Knoten erscheint im Gegensatz zu dem der Legende kaum lösbar. Das Land ist nach vier Jahrzehnten neoliberaler Wirtschaftspolitik zu gespalten und nach vier Jahren Populismus auch zu angespannt, als dass es sich hinter einem Präsident vereinen ließe. Sicher, Franklin D. Roosevelt konnte mit dem New Deal effektiv auf eine Weltwirtschaftskrise reagieren, aber seine Partei kontrollierte während seiner zwölfjährigen Präsidentschaft die meiste Zeit mit sehr großer Mehrheit beide Häuser des Kongresses. Auch Lyndon B. Johnson konnte das Civil-Rights-Gesetz von 1964 verabschieden, aber auch er hatte in beiden Häusern eine deutliche Mehrheit. Dieser Vorteil wird Joe Biden hingegen verwehrt bleiben. Die aktuellen Herausforderungen erscheinen jedoch gewaltig, sogar noch gewaltiger als die seiner großen Vorgänger, weil sich die Missstände über Jahrzehnte aufstauten und immer offener an den Tag treten. Natürlich wünschen wir ihm alles Gute. Selbst wenn die frisch gewählte Regierung nicht sämtliche von den oben dargestellten Problemen wird lösen können, ist die Hoffnung groß, dass mit dem Regierungswechsel eine deutliche Verbesserung gegenüber dem autokratischen Regierungsstil Trumps einhergehen wird. Und dennoch sollte jedem bewusst sein, dass diese 20 Herausforderungen auch weiterhin wie ein Damoklesschwert über der neuen Regierung, dem Land und der westlichen Welt schweben werden.

Prof. em. Dr. John Komlos war bis zu seiner Emeritierung Professor für Wirtschaftsgeschichte und -theorie an der Ludwig-Maximilians-Universität München.

Prof. Dr. Hermann Schubert ist Professor für Volkswirtschaftslehre an der International School of Management in Stuttgart.


Literatur

APM Research Lab Staff (2020), The Color of Coronavirus: Covid-19 Deaths by Race and Ethnicity in the U.S., November 12, https://www.apmresearchlab.org/covid/deaths-by-race (9. Dezember 2020).
Brynjolfsson, E. und A. McAfee (2014), The Second Machine Age. Work, Progress, and Prosperity in a Time of Brilliant Technologies.
Case, A. und A. Deaton (2020), Deaths of Despair and the Future of Capitalism, Princeton University Press.
DeSilver, D. (2017), 5 facts about the national debt, Pew Research Center, 17. August.
Federal Reserve Bank (2020a), 4th Quarter to 4th Quarter; series A939RX0Q048SBEA.
Federal Reserve Bank (2020b), series MPU4910013, https://fred.stlouis-fed.org (9. Dezember 2020).
Federal Reserve Bank (2020c), series MPU4900063, https://fred.stlouis-fed.org (9. Dezember 2020).
Federal Reserve Bank (2020d), series GFDEGDQ188S, https://fred.st-louisfed.org (9.12.2020).
Federal Reserve Bank (2020e): series NILFWJN, https://fred.stlouisfed.org (9. Dezember 2020).
Federal Reserve Bank (2020f): series LNS12032194, https://fred.stlouis-fed.org (9. Dezember 2020).
Federal Reserve Bank (2020g): series U6RATE, https://fred.stlouisfed.org (9. Dezember 2020).
Federal Reserve Bank (2020h): series UNRATE, https://fred.stlouisfed.org (9. Dezember 2020).
Federal Reserve Bank (2020i): series LES1252881900Q, https://fred.st-louisfed.org (9. Dezember 2020).
Federal Reserve Bank (2020j): series LES1252882800Q, https://fred.st-louisfed.org (9. Dezember 2020).
Federal Reserve Bank (2020k): series CPIAUCSL, https://fred.stlouisfed.org (9. Dezember 2020).
Gordon, R. (2016), The Rise and Fall of American Growth: The U.S. Standard of Living since the Civil War.
Komlos, J. (2015), Ökonomisches Denken nach dem Crash: Einführung in eine realitätsbasierte Volkswirtschaftslehre, Metropolis Verlag.
Komlos J Despair at Full Employment: The Urgency of a Fairer Labor Market Challenge: The Magazine of Economic Affairs 2019 61 5–6 363 386
Komlos J Schubert H Die Entwicklung sozialer Ungleichheit und ihre politischen Implikationen in den USA Wirtschaftsdienst 2019 99 3 216 223 10.1007/s10273-019-2421-0
Komlos J Schubert H Reaganomics — Wegbereiter des Trumpismus Wirtschaftsdienst 2020 100 1 64 71 10.1007/s10273-020-2563-0
Page, B. und M. Gilens (2017), Democracy in America? What Has Gone Wrong and What We Can Do About It.
Rogoff (2019), Government Debt Is Not a Free Lunch, Project Syndicate, 6. Dezember.
Rousseau, J.-J. (1762), Vom Gesellschaftsvertrag oder Prinzipien des Staatsrechtes.
Sachs, J. (2019), America’s Illusions of Growth, Project Syndicate, 14. Mai.
Sandel M Populism, liberalism, and democracy Philosophy & Social Criticism 2018 44 4 353 359 10.1177/0191453718757888
Summers L Demand Side Secular Stagnation American Economic Review 2015 105 5 60 65 10.1257/aer.p20151103
Taleb, N. (2007), The Black Swan: The Impact of the Highly Improbable.
Trump, D. (2019, 2020), Remarks by President Trump in State of the Union Address, 6. Februar 2019 und 4. Februar 2020.
Thukydides (um 411 v. Chr.), Geschichte des Peloponnesischen Krieges. U.S. International Trade in Goods and Services FT 900 (2020), Exhibit 1, https://www.census.gov/foreign-trade/Press-Release/2020pr/04/index.html#ft900 (30. Oktober 2020).
